# Expecting social punishment facilitates control over a decision under uncertainty by recruiting medial prefrontal cortex

**DOI:** 10.1093/scan/nsaa145

**Published:** 2020-11-10

**Authors:** Jaejoong Kim, Bumseok Jeong

**Affiliations:** Graduate School of Medical Science and Engineering, Korea Advanced Institute for Science and Technology (KAIST), Daejeon, 34141, Korea; Graduate School of Medical Science and Engineering, Korea Advanced Institute for Science and Technology (KAIST), Daejeon, 34141, Korea; KAIST Institute for Health Science and Technology and KI for Artificial Intelligence, KAIST, Daejeon, Korea

**Keywords:** social punishment, uncertainty, decision-making, computational modelling, dynamic causal modelling

## Abstract

In many decision-making situations, sub-optimal choices are increased by uncertainty. However, when wrong choices could lead to social punishment, such as blame, people might try to improve their performance by minimizing sub-optimal choices, which could be achieved by increasing the subjective cost of errors, thereby globally reducing decision noise or reducing an uncertainty-induced component of decision noise. In this functional magnetic resonance imaging (fMRI) study, 46 participants performed a choice task in which the probability of a correct choice with a given cue and the conditional probability of blame feedback (by making an incorrect choice) changed continuously. By comparing computational models of behaviour, we found that participants optimized their performance by preferentially reducing a component of decision noise associated with uncertainty. Simultaneously, expecting blame significantly deteriorated participants’ mood. Model-based fMRI analyses and dynamic causal modelling indicate that the optimization mechanism based on the expectation of being blamed would be controlled by a neural circuit centred on the right medial prefrontal cortex. These results show novel behavioural and neural mechanisms regarding how humans optimize uncertain decisions under the expectation of being blamed.

## Introduction

In our workplace, we make many decisions between options with uncertain values. If we fail to make a good decision, we might face a socially undesirable situation—such as being blamed by a boss. Therefore, although it could be hard to make a good decision because of uncertainty, we may become more deliberate and expend more effort to enhance the probability of optimal choice in this situation. We occasionally encounter this kind of stressful situation that might enhance decision performance at that moment but would make us feel bad.

The motivation to avoid negative outcomes might enhance task performance through several mechanisms, including increasing attention to the task (Engelmann, Damaraju, Padmala, & Pessoa, 2009) and enhancing working memory function (Krawczyk and D’esposito, 2013). Furthermore, the performance-enhancing effect by punishment has been thought to involve an increase in catecholamine level (Frankenhaeuser and Rissler, 1970). However, the behavioural and neural mechanisms that an agent uses to optimize an uncertain decision-making process to avoid a highly probable social punishment such as blame if their decision is wrong and how these kinds of socially stressful situation influences out mood have not been investigated.

A candidate mechanism of behaviour under uncertainty that could be controlled under threat to optimize behaviour is by reducing uncertainty-driven error in their action, and an unnecessary exploration could be a kind of that error. Exploration is the choice of an option that does not have maximum value among all options in the current state (Daw *et al.*, 2006; Payzan-LeNestour & Bossaerts, 2011), which could be either ‘directed’ to more uncertain options or ‘random’ (Gershman, 2018). Gershman and colleagues derived trial-by-trial uncertainties according to a Bayesian model of decision-making, and they showed that humans use both kinds of uncertainty-induced exploration (Gershman, 2018). Especially, they suggested that a total uncertainty induces a decision noise (uncertainty-induced decision noise [UDN]), which subsequently causes a random exploration (Gershman, 2018). Because both exploration strategies help an individual obtain information about the environment, it might also benefit a long-term cumulative reward in some situations (Agrawal and Goyal, 2012). However, if the uncertainties of every option are the same and an agent knows the outcomes of both selected and unselected options, exploration would be unnecessary because it does not maximize information gain or the reward; thus, choosing the option with the maximum value would be an optimal choice. We confined the situation regarding uncertain decisions to this type of situation to simplify our question about optimizing an uncertain decision under threat. In this situation, because uncertainties among options are the same, an uncertainty-directed exploration would not exist. However, an increase in total uncertainty would increase the decision noise, causing an unnecessary random exploration, which is an error in this case that might decrease the accuracy of the choice. Therefore, we hypothesized that controlling the sub-optimal choice (error) driven by UDN might help an agent make a more accurate choice when the wrong choice is likely to result in undesirable blame. Furthermore, we expected that blame in this situation would influence not only behaviour control under uncertainty but also people’s negative mood.

**Fig. 1.**
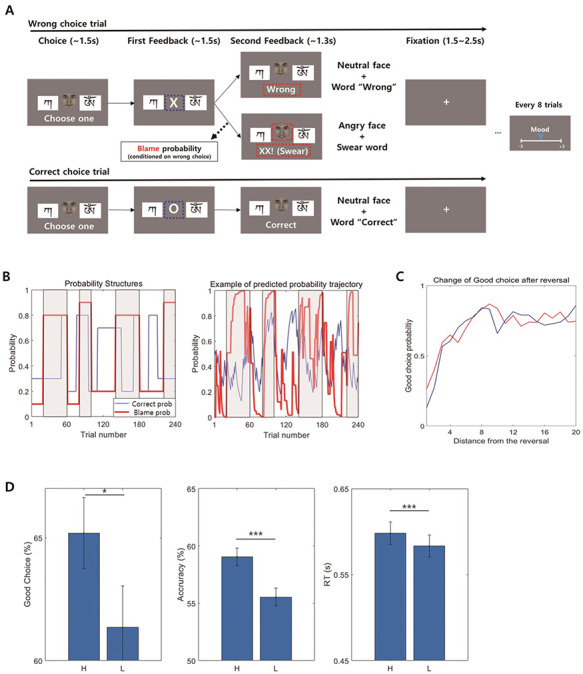
(A) Experimental paradigm. Two Tibetan character cues were presented, and cues were associated with a ‘correct’ outcome with the probability of *P* and 1−*P* each. This probability *P* changes between blocks of trials. If participants made a wrong choice, a blame composed of an angry face and swear words appeared probabilistically. (B) Probability structure of the task. Left: correct probability (Correct prob) and the conditional probability of blame given wrong choice (Blame prob) were changed between blocks of trials to change the participants’ estimation of uncertainty. Furthermore, there were high-blame blocks (grey-shaded area) and low-blame blocks. Right figure shows an example of belief trajectory regarding p(correct|cue) and p(blame|w) estimated from the CUDN model in one participant. The plot shows the similarity between the estimated belief trajectory and the designed probabilistic schedule. (C) A trajectory of good choice proportion for 20 trials after probability reversal. In both high- and low-blame blocks, good choice proportion kept increasing after the reversal. Furthermore, good choice probability was higher in the high-blame blocks than in the low-blame blocks early after reversals (about 10 trials after reversal). (D) Good choice proportion, accuracy and RT difference between high- and low-blame blocks. We compared the mean good choice proportion (left), accuracy (middle) and RT (right) between high-blame and low-blame blocks using a paired *t*-test. Good choice proportion was compared only in early trials after correct probability reversal. Participants showed increased good choice proportion, accuracy and RT within the high-blame blocks. The face used in this figure is different from the one that was used in the experiment to avoid using real human image in the figure (the face in this figure was generated by FaceGen Modeller (http://www.facegen.com/)).

To test this hypothesis, we designed a task with a choice between two options whose uncertainty changes during the task and both the outcomes of selected and unselected options are fully knowable (Figure 1A). Importantly, participants received blame with high or low probabilities when they made a wrong choice, and this conditional probability changed between blocks of trials. Note that, we used blame as an aversive stimulus that motivates one to avoid it which is similar to physical pain but encounters more frequently in our social life. However, our aim was not to compare both kinds of painful stimuli in this study. We predicted that participants would infer how likely they would be blamed by making a wrong choice and would control UDN to make a more accurate choice when blame was highly likely while their mood is negatively influenced. Note that the term ‘uncertainty’ used in our study indicates an ‘estimation uncertainty’ or ‘information uncertainty’ resulting from an insufficient estimation of the value (Payzan-LeNestour & Bossaerts, 2011). In our case, uncertainty is derived from an imperfect estimation of the probabilistic association between a cue and a ‘correct’ outcome (De Berker *et al.*, 2016). These hypothesis were tested by modelling participants’ behaviour and mood during the task using the novel hierarchal Bayesian reinforcement learning model. In this model, an agent infers both about (i) which option is likely to be correct and how such belief is uncertain and (ii) how it is likely to be blamed if one makes the wrong decision and these two kinds of inferences jointly influences the decision.

In order to identify a neural mechanism of such behavioural optimization under threat, model-based functional magnetic resonance imaging (fMRI) analyses and dynamic causal modelling (DCM) analysis (Friston *et al.*, 2003) were performed. A recent study suggested the medial prefrontal cortex (mPFC) as a candidate region for controlling strategic avoidance behaviour under threat (Mobbs *et al.*, 2007; Qi *et al.*, 2018). Furthermore, the mPFC implements slower, more controlled and deliberate decision-making during difficult choices (Cavanagh *et al.*, 2011) and drives strategy shifts (Schuck *et al.*, 2015). Therefore, we hypothesized that the neural circuit involving the mPFC would control the behavioural optimization process under the expectation of being blamed. Especially, we expected that such a neural circuit would also involve the rostrolateral prefrontal cortex (rlPFC), which is related to an uncertainty-driven exploration in the previous literature (Badre *et al.*, 2012).

## Materials and Methods

### Participants

Forty-six participants (32 males and 14 females, mean age of 22.61 ± 3.61 years) from the Korea Advanced Institute for Science and Technology (KAIST) volunteered for this experiment. Details of participants’ information can be found in the Supplementary data.

### Ethics statement

All participants provided written informed consent to participate in the experiment based on sufficient explanation about the study (including blame). The study was approved by the KAIST Institutional Review Board in accordance with the Declaration of Helsinki.

### Experimental task

Every participant completed 240 trials of a choice task in which the goal was to acquire the ‘correct’ outcome as many times as possible. In every trial, two Tibetan character cues were presented, and participants were asked to choose one (Figure 1A). Cues were probabilistically associated with either a ‘correct’ outcome or a ‘wrong’ outcome, and this correct probability was reciprocal such that

$$\begin{equation*}{\rm{Correct\,\, probability\,\, of\,\, cue}}\,\,{\rm{1}}\,\,{\rm{ = }}\,\,{\rm{1-Correct\,\, probability\,\, of\,\, cue}}\,\,2,\end{equation*}$$

which was explicitly conveyed to participants to make the uncertainties about the correct probabilities for both cues to be equal, thus minimizing directed exploration. This probability was changed every 10–50 trials between 20/30% and 80/70% to change the participants’ estimation of the uncertainty of association probability between the cue and the correct choice (Figure 1B). They failed to make a good choice (designed to have a higher probability of correct) right after probability reversal. However, their good choice probability increased through learning (Supplementary Figure S1). Independent with this correct probability, participants had to think about whether they might face a socially undesirable situation if they fail to make the right decision in the current trial. Particularly, if they made a wrong choice, they were probabilistically being blamed with an angry face and swear words. We called this type of feedback blame, and we instructed participants to regard this feedback as blame in the context of social situation, such as a blame given by their boss or superior (e.g. ‘Please try to imagine as vividly as possible that your boss yells at you because you made a wrong choice’). Participants sufficiently practised imagining this situation before starting the experiment (Figure 1A). Importantly, we varied the conditional probability of the appearance of the blame feedback when participants made an incorrect choice block by block with a range from 20 to 40 trials. Thus, during some blocks of the task, the conditional probability of blame feedback was very high when the choice was wrong (80% or 90%; we designated these blocks ‘high-blame’ blocks, grey-shaded area of Figure 1B), while in the other blocks, this conditional probability was low (10% or 20%; we designated these blocks ‘low-blame’ blocks). Additionally, to assess the mood of each trial, participants were instructed to rate their mood on a Likert scale ranging from −3 to 3 every eight trials after receiving secondary feedback. A total of 30 ratings were acquired from each participant. We expected that participants would implicitly or explicitly calculate the conditional probability of being blamed by making a wrong choice (we represent this subjective conditional probability of being blamed for a wrong choice of each participant as ‘p(blame|w)’), and this calculation would influence participants’ decision-making, such as encouraging participants to make more deliberate and careful choices and mood, which was revealed in the results of the post-experimental survey (Supplementary Table S1).

## Behaviour analyses

### Testing effect of blame expectation on making a better choice under uncertainty

We first examined whether people made better choices in the trials that a wrong choice is highly likely to result blame. To define a better choice, we defined a ‘good choice’ and ‘accurate choice’. In each block, there was a cue that was designed to have a higher correct probability than other cue and choosing this better cue was defined as a ‘good’ choice of that block. For example, if a cue1 was designed to have 70% correct probability in one block (thus cue2 has 30% correct probability), that cue is the good choice of that block. An ‘accurate’ choice was defined as a choice that resulted in actual ‘Correct’ feedback at each trial. Because ‘Correct’ feedback was probabilistic given the choice, people might not get ‘Correct’ feedback even if they made a good choice. We compared a good choice proportion and mean accuracy between the high-blame blocks and low-blame blocks using the paired *t*-tests. Furthermore, because we hypothesized that an effect of blame expectation would depend on uncertainty (decreasing UDN), we also compared the good choice proportion in trials expected to have high uncertainty. Considering that uncertainty is known to be maximized after probability reversal and reduced until the next reversal because of the learning (De Berker *et al.*, 2016), we only used the first 10 trials after a reversal of the correct probability in this test. Additional behaviour analysis can be found in the Supplementary data.

### Computational modelling of an effect of blame belief on sub-optimal decision

**Fig. 2.**
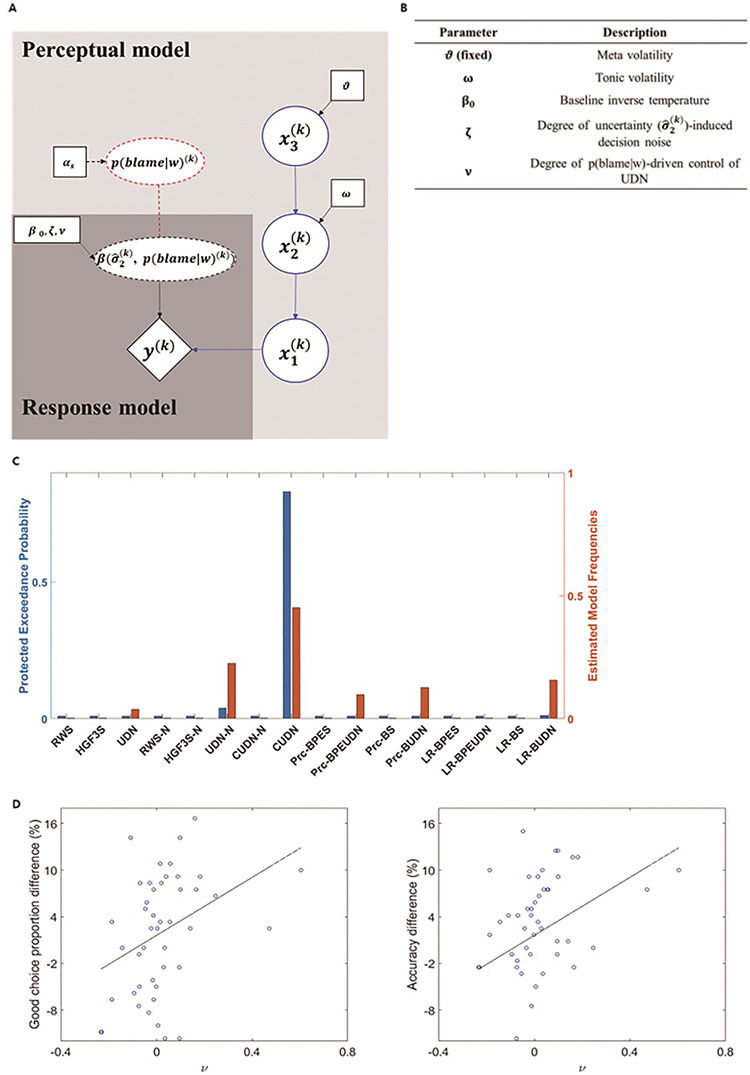
(A) Graphic description of the computational model of behavioural optimization under the expectation of being blamed. The CUDN model was composed of the following parts: the perceptual model and response model. The perceptual model is composed of two parallel learning systems—learning p(correct|cue) by the three-level hierarchical Gaussian filter (HGF) model (blue) and learning p(blame|w) by the Rescorla–Wagner (RW) model (red dashed circle). Importantly, in the response model, ${y^{\left( k \right)}}$ represents a choice at trial k, and the $\beta $, which is the inverse temperature of the Softmax function, is a function of an estimation uncertainty $(\hat \sigma _2^{\left( k \right)})$ and $ p{(blame|w)^{\left( k \right)}}$ such that $\hat \sigma _2^{\left( k \right)}$ induces decision noise and $ p{(blame|w)^{\left( k \right)}}$ suppresses UDN. In this graphic representation, a deterministic node and relationship are represented as dashed circles and dashed lines, respectively, while solid circles and lines represent a stochastic node and relationship. (B) Parameters of the CUDN model. In the function $\beta \left( {\hat \sigma _2^{\left( k \right)}, p{{(blame|w)}^{\left( k \right)}}} \right)$, parameter $\zeta $ determines the participants’ degree of UDN, and $\nu $ determines the participants’ degree of suppression on UDN as p(blame|w) increases. (C) Bayesian model selection results. The results of the RFX-BMS show that the CUDN model fits the participants’ behaviour better than other models (PEP = 0.831). (D) Correlation between ${\bf{\it{\nu }}}$ and good choice/accuracy enhancement. The results of correlation analysis showed that the participants with large $\nu $ showed enhanced good choice proportion (left) and accuracy (right) in high-blame blocks. Note that these significant correlations also existed after removing points with values outside of 2 s.d. (rightmost 2 points) from the mean (all *P* < 0.05).

After showing that participants made better decision under uncertainty when a wrong choice is highly likely to result a blame, we designed explicit computational models that can explain the internal process of computing beliefs regarding conditional probability of the blame (p(blame|w)), and this belief subsequently influences participants’ decision. We hypothesized that uncertainty would increase the decision noise that increases a chance of sub-optimal choice and p(blame|w) would decrease this UDN, meaning an interaction effect between p(blame|w) and uncertainty on decision noise. Note that sub-optimal choice here was defined with respect to an internal belief (choosing an option that is believed to have lower correct probability than other options), making it different from a bad choice. A computational model explains this process, which was named as control of uncertainty-induced decision noise (CUDN) model (Figure 2A). In this model, the parameter $\nu $ determined the degree of suppression of UDN as p(blame|w) increases. Therefore, if $\nu $ is large for some participants, those participants greatly suppress UDN when p(blame|w) is high compared with when p(blame|w) is low and vice versa (Supplementary Figure S3). We fitted the CUDN model to the participants’ responses and compared this model with 15 other models (Supplementary Table S4) with random-effect Bayesian model selection (RFX-BMS) (Stephan *et al.*, 2009; Daunizeau *et al.*, 2014). A summary of the 16 models is provided in Supplementary Table S4. Other details of the computational modelling can be found in the Supplementary data.

### Model-based fMRI analysis

We performed a model-based fMRI analysis based on the CUDN model to investigate the neural substrate of suppression of UDN under p(blame|w). We performed parametric modulation analyses using the trial-by-trial trajectories of model variables of interest for each subject as parametric modulators of a first-level general linear model (GLM) in Statistical Parametric Mapping 12 (SPM12) (Penny *et al.*, 2011). Variables of interest at the onset of the cue included trial-by-trial p(blame|w) and uncertainty that influenced decision noise in the CUDN model. Moreover, we included the trial-by-trial subjective value of the sub-optimal option (that is, subjective correct probability (p(correct|cue)) of the sub-optimal option), which induces an error (sub-optimal choice). Blame prediction error (BPE) regressor was also added as a parametric modulator at the onset of secondary feedback. Details of the methods regarding fMRI acquisition, pre-processing and parametric modulation analyses can be found in the Supplementary data.

### DCM

In the parametric modulation analysis, we found that the bilateral mPFC was robustly activated by p(blame|w), which is consistent with our hypothesis. We expected that the mPFC would be a region that controls the UDN according to the p(blame|w), and this control process would be implemented by a dynamic change of interaction between the mPFC and the region processing sub-optimal option such as the rlPFC. To test this hypothesis, we first confirmed that their connection varies by the p(blame|w) using the psychophysiological interaction (PPI) (Friston *et al.*, 1997) and investigated more detailed dynamics and their relationship with UDN control process using the DCM analysis (Friston *et al.*, 2003). Details of the methods regarding PPI and DCM analyses can be found in the Supplementary data.

## Results

### Behaviour analyses

#### Expectation of being blamed facilitates better performance by deliberation.

Overall, people successfully followed our sophisticated probabilistic design (68.2% of good choice proportion, significantly better than chance (50%): *t*[45.00] = 15.78, *P* < 0.001), this performance was similar but slightly lower to the performance of ideal Bayesian learner (70.1%, Supplementary data). They made more good choice in the high-blame blocks only with a marginal significance (mean good choice proportion = 0.692 *vs* 0.673, *t*[45] = 1.76, *P* = 0.085, confidence intervals [CIs]: 0.00 to 0.04, *d* = 0.3; two-tailed paired *t*-test; Figure 1D, left). However, when compared using only uncertain trials, increment of participants’ good choice proportion was small, but significant in the high-blame blocks (0.652 *vs* 0.614, *t*[45] = 2.02, *P* = 0.049, CIs: 0.000 to 0.077, *d* = 0.298, paired *t*-test), suggesting that an effect of blame expectation on promoting good choices might depend on the uncertainty level. Participants also choice with 57.3% accuracy (significantly better than chance, [45] = 11.45, *P* < 0.001) and they also more accurate choice in the high-blame blocks compared with the low-blame blocks (mean accuracy = 0.591 *vs* 0.555, *t*[45] = 4.09, *P* < 0.001, CIs: 0.02 to 0.05, *d* = 0.6; Figure 1D, middle). Moreover, the mean response times (RTs) of the high-blame blocks were significantly longer than those of the low-blame blocks (mean RT = 598 ms *vs* 583 ms, *Z* = 3.95, *P* < 0.001, Wilcoxon signed-rank test; Figure 1D, right). Based on these results, we expected that participants made better decisions under uncertainty via deliberation when p(blame|w) was high.

#### Computational modelling: expecting blame optimizes decision under uncertainty while negatively influences mood.

The CUDN model was selected with a protected exceedance probability (PEP) of 0.831 among 16 candidate models, suggesting that participants were able to control the influence of subjective uncertainty on their decision depending on the belief of how they are likely to be blamed if they make an incorrect choice (Figure 2C). Furthermore, the degree of suppression on UDN under high p(blame|w), which was parameterized by $\nu $ had significant positive correlation with both increase in the good choice proportion (Spearman’s ${\rm{\rho }}$ = 0.384, *P* = 0.008, Figure 2D) and accuracy (Spearman’s ${\rm{\rho }}$ = 0.306, *P* = 0.016, Figure 2E) in high-blame blocks, meaning that participants were able to make better decision under social threat by suppressing UDN. Although p(blame|w) enabled a flexible control of the UDN, this kind of belief significantly decreased the mood of participants (Supplementary data). Although ν well explained individual differences of an effect of p(blame|w) on controlling UDN, the group average of the ν was not significantly different from the 0 (*t* = 0.93, *P* = 0.357). Finally, because the computational modelling results can be susceptible to the prior parameter setting, we performed the susceptibility analyses on the prior parameter setting of the ν. In all settings, the PEP of the CUDN model was greater than other models. However, this analysis showed that the PEP of the CUDN model changed according to the prior parameter settings and in some settings it was not sufficiently large enough (Daunizeau *et al.*, 2014) (e.g. PEP = 0.531 in setting of prior mean = 0, prior variance = 1, Supplementary data), which could be the limitation of our computational modelling.

### Model-based fMRI analysis

#### The bilateral mPFC is recruited by an expectation of blame, and the bilateral rlPFC is involved in processing of sub-optimal option.

**Fig. 3.**
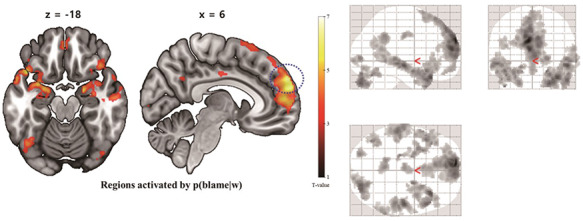
Results of the parametric modulation analysis by p(blame|w). MPFC, PCC (centre) and hippocampus (left), were activated by p(blame|w) (cluster-level whole brain FWE-corrected *P*-value < 0.001, CDT = 0.001). The blue dashed circle denotes the right mPFC used in the DCM as a region of interest (ROI).

**Fig. 4.**
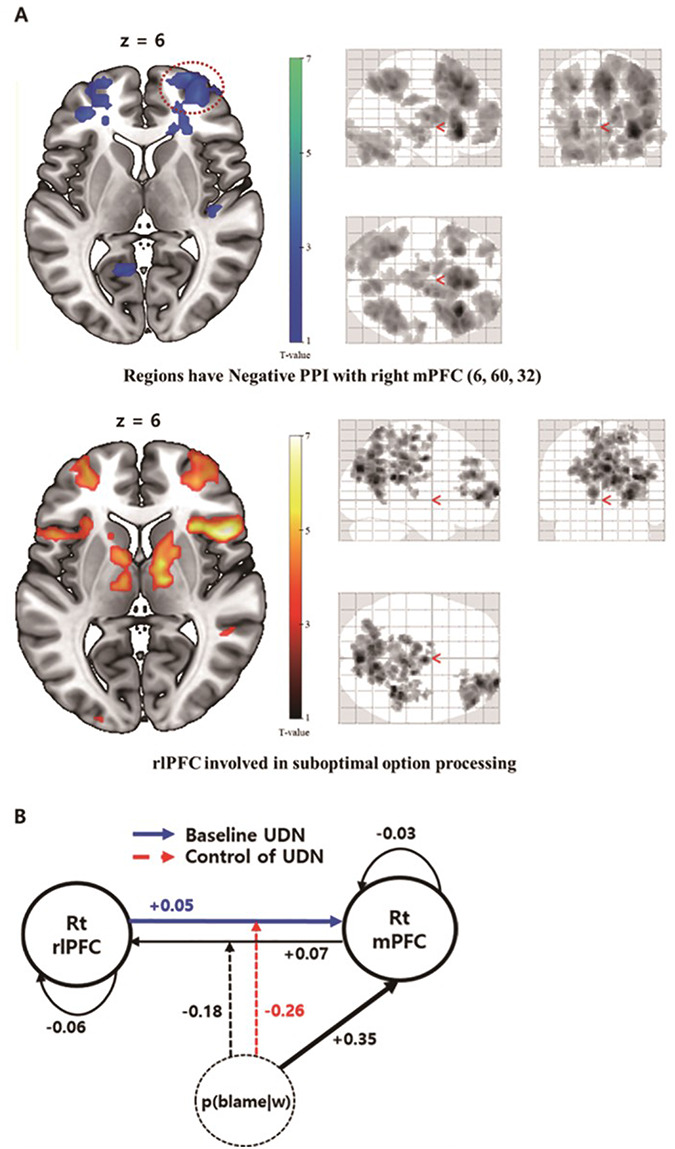
(A) Modulation of connectivity between the right mPFC and other regions by a blame expectation. The results of the PPI analysis (upper figure) suggested that functional connectivity between the right mPFC and right rlPFC was negatively modulated as p(blame|w) increased (red dashed circle, cluster-level whole brain FWE-corrected *P*-value = 0.039, CDT = 0.05). This rlPFC cluster had large overlap with the rlPFC clusters involved in the processing of the sub-optimal option (lower figure, cluster-level whole brain FWE-corrected *P*-value < 0.001, CDT = 0.001). (B) DCM models explaining dynamics between right mPFC and rlPFC. The winning model showed the bidirectional fixed connections between the right mPFC (blue dashed circle in Figure 3) and rlPFC (red dashed circle in Figure 4A). Both connections were modulated by p(blame|w) and p(blame|w) also acted as a driving input to the mPFC. The fixed connection from the right rlPFC to the mPFC (blue arrow) was related to the degree of UDN. Moreover, p(blame|w) acted as a driving input to the right mPFC, and the effective connectivities from the right mPFC to the rlPFC as well as from the right rlPFC to the mPFC were modulated by p(blame|w) (red dashed line). The modulation of effective connectivity from the right rlPFC to the mPFC was related to the degree of UDN control via p(blame|w).

In the parametric modulation analyses of the p(blame|w), robust activation of a bilateral mPFC cluster extending to the lateral prefrontal cortex (lPFC) and dorsal anterior cingulate cortex (dACC) and the bilateral temporal pole cluster extending to the bilateral amygdala and hippocampus was observed (cluster-level whole brain familywise-error (FWE)-corrected *P*-value < 0.001 in both clusters, cluster-defining threshold [CDT] *P* = 0.001, uncorrected, Figure 3 and Table 1). Interestingly, in our additional conjunction analyses (Supplementary data), there was an overlapping activation within ventromedial prefrontal cortex (vmPFC) between p(blame|w) and subjective value difference between chosen and unchosen options (Palminteri *et al.*, 2015)—where an increase of such differences are related to an increase of optimal choice (cluster-level whole brain FWE-corrected *P* < 0.001, CDT *P* = 0.001, uncorrected, Supplementary Figure S9A). Therefore, we suspected that this overlap within vmPFC might support the role of p(blame|w) in decreasing decision noise. Furthermore, both p(blame|w) and the variables related to the cognitive control load (decrease of absolute value difference or RT, Supplementary data) activated dACC (cluster-level whole brain FWE-corrected *P* = 0.008, CDT *P* = 0.001, uncorrected, Supplementary Figure S9B) and lPFC (cluster-level whole brain FWE-corrected *P* = 0.005, CDT *P* = 0.001, uncorrected, Supplementary Figure S9C) in the conjunction analyses. Note that both regions are previously known as a part of cognitive control network (Shenhav *et al.*, 2013), and thus, we suspect that this might be related to an increase of deliberation according the level of p(blame|w). Next, a parametric modulator regarding the value (subjective correct probability) of the sub-optimal option co-varied with the blood-oxygen-level-dependentsignal of the bilateral rlPFC (cluster-level whole brain FWE-corrected *P* < 0.001, CDT *P* = 0.001, uncorrected, Figure 4A, Supplementary Figure S6 and Table S7), which regions were engaged in uncertainty-driven exploration in a previous study (Badre *et al.*, 2012). More detailed results of parametric modulation analysis are provided in the Supplementary data.

**Table 1.** Regions activated by p(blame|w)

Cluster (CDT *P* = 0.001, uncorrected)	Cluster *P*-value (whole brain FWE corrected)	No. of voxels	Region name (AAL)	MNI coordinates (x, y, z)	Voxel *Z*-value (uncorrected *P*-value)
mPFC	<0.001	4556	Frontal_Sup_Medial_L	−8, 62, 28	6.65 (<0.001)
			Frontal_Sup_Medial_R	6, 60, 32	5.27 (<0.001)
Right TP/hippocampus/amygdala	<0.001	1156	Temporal_Pole_Mid_R	−4, −46, 35	5.51 (<0.001)
			Hippocampus_R	20, 4, 18	4.93 (<0.001)
Left TP/hippocampus/amygdala	<0.001	1368	Temporal_Pole_Sup_L	−44, 16, −40	4.95 (<0.001)
PCC	<0.001	407	Cingulate_Mid_L	−4, −46, 34	4.61 (<0.001)
			Cingulate_Post_L	−12, −46, 30	4.50 (<0.001)
Right MTG/STG	<0.001	1120	Temporal_Mid_R	52, −18, −10	5.47 (<0.001)
Left MTG/STG	<0.001	814	Temporal_Mid_L	−56, −28, −6	5.13 (<0.001)
Left MTG	0.003	210	Temporal_Mid_L	−42, −60, 20	4.05 (<0.001)
dACC/MCC	0.007	178	Cingulate_Mid_L	−2, −18, 34	4.35 (<0.001)
Cerebellum	<0.001	817	Cerebelum_Crus2_R	24, −86, −36	5.31 (<0.001)
Cerebellum	0.009	166	Cerebelum_Crus1_L	−42, −68, −24	4.76 (<0.001)
Cerebellum	0.012	157	Cerebelum_Crus2_L	−22, −86, −34	4.66 (<0.001)

AAL, automated anatomical labeling; TP, temporal pole; MTG, middle temporal gyrus; STG, superior temporal gyrus.

#### Blame expectation negatively modulates effective connectivity from the right rlPFC to the right mPFC to suppress the UDN.

In the PPI analysis, we found out that functional connectivity between right mPFC and right rlPFC was negatively modulated by p(blame|w) (cluster-level whole brain FWE-corrected *P* = 0.039, CDT *P* = 0.05, Figure 4A and Supplementary Table S8) and the degree of negative modulation was significantly correlated with $\nu $ (Spearman’s ${\rm{\rho }}$ = —0.34, *P* = 0.029). To clarify the specific neural dynamics and to identify how those dynamics account for the control of UDN under blame expectation, we performed DCM analysis between two regions. In the RFX-BMS among 16 DCM models considering all possible interaction patterns between 2 regions, model 7 (Figure 4B), which includes a driving input to the right mPFC by p(blame|w), a bidirectional fixed connection between two regions and modulation of both connections by p(blame|w), was selected with a PEP of 0.999. We conducted a robust linear regression analysis using the parameter $\nu $ as a dependent variable and two effective connectivities, one from the right mPFC to the right rlPFC and another from the right rlPFC to the right mPFC in model 7, as independent variables to identify which direction of modulation by p(blame|w) was related to the suppression of UDN. Only the effective connectivity from the right rlPFC to the right mPFC negatively influenced $\nu $ (${\rm{beta}}$ = −0.46, *t*[29] =  −3.09, *P* = 0.004, CIs: −0.75 to −0.17, *d* = −0.5), whereas the effective connectivity in the opposite direction did not (${\rm{beta}}$ = −0.2, *t*[29] =  −1.33, *P* = 0.193, CIs: −0.49 to 0.09, *d* = −0.2). Furthermore, because the modulation from the baseline ‘fixed’ connectivity was related to the modulation of UDN by p(blame|w), we suspected that the fixed connection from the right rlPFC to the right mPFC might be related to the UDN and the modulation of this connection is related to the control of UDN. To test this hypothesis, we performed a robust linear regression analysis using the parameter ζ, which is related to the UDN as the dependent variable and the fixed connection from the right rlPFC to the right mPFC as an independent variable, which showed a positive relationship between two variables (beta = 0.24, *t*[30] = 2.08, *P* = 0.047, CIs: 0.01 to 0.46, *d* = 0.4, Figure 4B). In summary, we showed that the UDN is related to the fixed connection from the right rlPFC to the right mPFC and that the modulation of this connection via p(blame|w) is related to UDN suppression.

## Discussion

Under the condition that a wrong decision leads to severe blame by another, we must regulate ourselves to make better decisions. In such situation, participants enhanced their ability to make better choices under uncertainty through the suppression of UDN. This means that when one is already confident about the best action, then an expectation of being blamed has little effect—however, when one is unsure, then blame tends to increase optimal choices by reducing random exploration. However, expecting such blame with high probability impaired participants’ mood. Furthermore, fMRI analyses, including DCM analyses, revealed that a neural mechanism underlying this behavioural tendency is related to the suppression of connectivity from the right rlPFC to the mPFC as p(blame|w) increases, where the right rlPFC was engaged in processing the sub-optimal option and the right mPFC was activated by p(blame|w).

Our study improves our understanding of the behavioural and neural mechanisms of optimal decision-making strategies to avoid aversive outcomes expected for incorrect decisions in a social context and how such kind of stressor influences our mood. From a behavioural perspective, when punishment or loss is expected with lower performance, participants enhance their performance by enhancing working memory function (Krawczyk and D’esposito, 2013) or inhibitory control mechanisms (Simões‐Franklin *et al.*, 2010). However, to the best of our knowledge, few studies have proposed the computational mechanisms that are involved in optimizing uncertain decisions under punishment expectations based on explicit computational models of behaviour. In the present study, using the CUDN model, we successfully showed that participants increased good and accurate choices when a conditional probability of blame given a wrong choice was high through the flexible control of UDN.

In many cases, random exploration might be beneficial for maximizing a long-term expected reward because it helps the individual obtain information about uncertain options (Agrawal and Goyal, 2012; Payzan-LeNestour & Bossaerts, 2011). However, in situations such as our task, where there is no more information gain by choosing one option over another, random exploration would become a choice that might decrease the accuracy of the choice. We modelled this effect of expectation of being blamed on uncertainty-driven sub-optimal choices using a CUDN model in which an uncertainty increases decision noise and p(blame|w) controls this UDN; this model explained participants’ behaviours better than other models. An interesting point regarding models where p(blame|w) had a negative value is that p(blame|w) naturally decreased the decision noise, regardless of the amount of uncertainty, which is different from the CUDN model in which only UDN is influenced by p(blame|w). Therefore, another point recognized from the Bayesian model selection results is that p(blame|w) likely controls the decision noise in a manner proportional to a degree of uncertainty but not in an uncertainty-independent manner. Because the decision noise increases with uncertainty, the requirement for the suppression of UDN to make an optimal choice is higher when high uncertainty exists. Furthermore, based on the results of the post-experimental survey, we recognized that participants made more deliberate, effort-driven decisions under threat, resulting in the suppression of (uncertainty-driven) decision noise. Thus, we surmise that the suppression of decision noise might require a mental effort that is costly to exert (Botvinick and Braver, 2015; Shenhav *et al.*, 2017). Therefore, we speculated that a balance between the cost of deliberation and the need to suppress decision noise to make an optimal choice under uncertainty might increase the efficiency of the suppression of UDN, particularly in a volatile environment in which an uncertainty level continuously changes, such as in our task.

After confirming that p(blame|w) controlled the UDN, we revealed that the suppression of UDN enabled the participant to make a better choice by showing a positive correlation between increased good choice proportion and accuracy in the high-blame blocks and the model parameter $\nu $. Therefore, our behavioural results suggest that when a wrong choice is likely to result in an aversive outcome under the uncertain decision-making situation used in our task, an agent tries to make a better choice by suppressing UDN to some degree. However, although ν well explained individual differences regarding the effect of p(blame|w) on controlling UDN, it seems that this parameter alone is insufficient to explain this effect. Considering that group average of ν was not significantly different from the 0 despite increased good/accurate choice in high-blame blocks, we speculate that this effect could be implemented by a whole CUDN model including interactions between ν other parameters. For example, because an increase of optimal choice depends on decreasing UDN, an effect of p(blame|w) on controlling UDN would be affected also by the ζ. Note that ζ determines the degree of decision noise induced by an uncertainty. This point is a limitation of our study and should be investigated in future studies. Additionally, we also have shown that an inference regarding a conditional probability of the blame significantly impaired participants’ mood. People suffer from abusive supervision are at high risk of affective disorder (Tepper *et al.*, 2007). We speculate that people who are frequently exposed to abusive supervision similar to our task such that their supervisor forces them to make good decision under uncertainty with social punishment would be more likely to evolve an affective disorder by an accumulation of a daily negative mood induced by an aversive outcome expectation by their wrong decision.

We then identified the neural mechanism underlying the expectation of blame and the suppression of the UDN by p(blame|w). The regions involved in processing p(blame|w) included the mPFC, hippocampus and posterior cingulate cortex (PCC), which are similar to the regions involved in the ‘cognitive fear circuitry’ (Qi *et al.*, 2018). The suggested role for this circuit was strategic avoidance of a threat (Qi *et al.*, 2018), and the authors mentioned that the mPFC is likely involved in selecting defensive response strategies (Qi *et al.*, 2018). This region contains ‘strategy-selective’ cells that protect against threats, as reported in an animal study (Halladay and Blair, 2015). Moreover, the mPFC is related to internally driven strategy shifts (Schuck *et al.*, 2015). Consistent with these studies, subsequent DCM analyses revealed the involvement of the mPFC in the control of UDN. Especially, neural dynamics involving the right mPFC and rlPFC were related to the control of UDN via p(blame|w). Two regions had bilateral fixed connections, and these connections were negatively modulated according to the p(blame|w). Importantly, among these two directional connections, only the connection from the right rlPFC to the right mPFC was relevant to the control of UDN, such that the UDN itself was related to the fixed connection from the right rlPFC to the right mPFC, and the control of the UDN was related to the modulation of this connection via p(blame|w).

Based on these results, we propose a possible neural ‘gate’ model regarding the control of the UDN based on p(blame|w) that fits with the structure of the CUDN model. In the CUDN model, given a fixed value of the sub-optimal option, (i) uncertainty increased the probability of choosing the sub-optimal option and (ii) p(blame|w) decreased such effect of uncertainty (represented by the decision noise modulation in the model). Similarly, in the DCM analyses, (i) influence of uncertainty on decision temperature (UDN) was related to the strength of fixed connectivity from rlPFC to mPFC and (ii) p(blame|w) decreased the connectivity from rlPFC to mPFC, which was related to a p(blame|w)’s effect on decreasing UDN. (iii) p(blame|w) increased the mPFC activity. From these information, we focused on how p(blame|w) information can modulate the connectivity from rlPFC to mPFC while activating the right mPFC. We suggest that (i) the UDN information is encoded in the fixed connection from the right rlPFC to the mPFC. (ii) If p(blame|w) increases, p(blame|w) information is conveyed to the right mPFC and activates this region. (iii) Using this p(blame|w) information, the mPFC regulates the flow from the right rlPFC to the mPFC to control the UDN. This gate model could explain the concurrent activation of the mPFC and the negative modulation of the connection from the right rlPFC and the mPFC based on p(blame|w). This gate model not only corroborates our behavioural and fMRI analyses but also is consistent with previous studies suggesting that the mPFC controls strategic avoidance behaviour under threat (Qi *et al.*, 2018). Furthermore, a previous study suggested that the mPFC controls the slower and deliberate decision-making associated with difficult choices by modifying the decision threshold of the drift-diffusion model via an interaction with the sub-thalamic nucleus (Cavanagh *et al.*, 2011). Especially, increasing of decision threshold increases the RT and reduces the choice noise (Reddi *et al.*, 2003), which is effect similar to that of an effect of p(blame|w). Therefore, it could be possible that a ‘gate’ in the mPFC controlled by p(blame|w) might correspond to an increase of decision threshold.

One notable point is that whether our result could be generalizable to non-social punishment such as physical pain. Because we did not perform a similar experiment using a non-social punishment (e.g. physical pain or monetary loss), it is difficult to determine whether this effect is universal to all types of punishment or specific to blame. However, the results of this study allow us to infer the similarity and dissimilarity of the blame with other non-social stimuli. For example, if the expectation of blame is similar to that of loss, we would expect that the p(blame|w) would contribute as a negative value; however, this result was not the case in the behavioural modelling section. Furthermore, previous studies have shown that physical and social pain are similar in both emotional response and saliency as well as share a similar neural representation (Eisenberger, 2012). Moreover, we observed a similar between updating blame expectation and updating of physical pain expectation, which we discussed in the above section (Roy *et al.*, 2014). Therefore, we hypothesize that the effect of expecting blame might be more similar to that of expecting physical pain than to that of expecting a monetary loss. However, this hypothesis is only speculative, and an experiment using non-social stimuli might help us to identify both the similar and different neural and behaviour mechanisms that underlie the optimization of behaviour under social and non-social threats. Finally, one should note that one limitation of our computational modelling was that the result was susceptible to the selection of prior parameters, which could be resulted from the complexity of the CUDN model.

In conclusion, we identified one strategy for optimizing uncertain decision-making under a threat and the underlying neural mechanism. Because there was no benefit of the sub-optimal choice in our task, the suppression of UDN under the blame expectation helped participants to make better decisions in those situations, and this phenomenon was successfully modelled using the CUDN model. On the other hand, an expectation of being blamed deteriorated participants’ mood. The implementation of this behavioural optimization strategy was related to the suppression of effective connectivity from the right rlPFC to the right mPFC as p(blame|w) increased. These results added one novel neural mechanism of a brain region related to processing threat that actually interacted with other decision-making-related regions to avoid a threatening outcome.

Because we addressed only one optimization mechanism under particular conditions, where directed exploration is absent or minimal, an extension of our research to determine how directed exploration is influenced in this situation would be interesting. Based on recent findings that people became more ‘myopic’ under the threat (Korn and Bach, 2019), we speculate that directed exploration would also be reduced by blame expectation and it could explain reduced creativity under the threat. Finally, from the perspective of computational psychiatry, an investigation of the optimization behaviour of our task in patients with psychiatric conditions, such as autism and psychopathologies, would be interesting to quantify their lack of an ability to expect social responses and utilize adaptive behaviours (Sevgi *et al.*, 2016).

## Supplementary Material

nsaa145_Supp

## Data Availability

Source codes for computational models of behaviour are accessible at the 10.6084/m9.figshare.8157896. Other data from the current study are available from the corresponding author upon reasonable request.

## References

[R1] Agrawal S., Goyal N. (2012). Analysis of Thompson sampling for the multi-armed bandit problem. Paper presented at the Conference on Learning Theory.

[R2] Badre D., Doll B.B., Long N.M., Frank M.J. (2012). Rostrolateral prefrontal cortex and individual differences in uncertainty-driven exploration. *Neuron*, 73(3), 595–607. 22325209 10.1016/j.neuron.2011.12.025 PMC3285405

[R3] Botvinick M., Braver T. (2015). Motivation and cognitive control: from behavior to neural mechanism. *Annual Review of Psychology*, 66, 83–113. 10.1146/annurev-psych-010814-015044 25251491

[R4] Cavanagh J.F., Wiecki T.V., Cohen M.X., et al. (2011). Subthalamic nucleus stimulation reverses mediofrontal influence over decision threshold. *Nature Neuroscience*, 14(11), 1462. 10.1038/nn.2925 PMC3394226 21946325

[R5] Daunizeau J., Adam V., Rigoux L. (2014). VBA: a probabilistic treatment of nonlinear models for neurobiological and behavioural data. *PLoS Computational Biology*, 10(1), e1003441. 10.1371/journal.pcbi.1003441 PMC3900378 24465198

[R6] Daw N.D., O’doherty J.P., Dayan P., Seymour B., Dolan R.J. (2006). Cortical substrates for exploratory decisions in humans. *Nature*, 441(7095), 876. 10.1038/nature04766 PMC2635947 16778890

[R7] De Berker A.O., Rutledge R.B., Mathys C., et al. (2016). Computations of uncertainty mediate acute stress responses in humans. *Nature Communications*, 7, 10996. 10.1038/ncomms10996 PMC4820542 27020312

[R8] Eisenberger N.I. (2012). The pain of social disconnection: examining the shared neural underpinnings of physical and social pain. *Nature Reviews Neuroscience*, 13(6), 421. 10.1038/nrn3231 22551663

[R9] Engelmann J.B. (2009). Combined effects of attention and motivation on visual task performance: transient and sustained motivational effects. *Frontiers in Human Neuroscience*, 3, 4. 10.3389/neuro.09.004.2009 PMC2679199 19434242

[R10] Frankenhaeuser M., Rissler A. (1970). Effects of punishment on catecholamine release and efficiency of performance. *Psychopharmacologia*, 17(5), 378–90. 5522998 10.1007/BF00403809

[R11] Friston K., Buechel C., Fink G., Morris J., Rolls E., Dolan R.J. (1997). Psychophysiological and modulatory interactions in neuroimaging. *Neuroimage*, 6(3), 218–29. 9344826 10.1006/nimg.1997.0291

[R12] Friston K.J., Harrison L., Penny W. (2003). Dynamic causal modelling. *Neuroimage*, 19(4), 1273–302. 12948688 10.1016/s1053-8119(03)00202-7

[R13] Gershman S.J. (2018). Deconstructing the human algorithms for exploration. *Cognition*, 173, 34–42. 29289795 10.1016/j.cognition.2017.12.014 PMC5801139

[R14] Halladay L.R., Blair H.T. (2015). Distinct ensembles of medial prefrontal cortex neurons are activated by threatening stimuli that elicit excitation vs. inhibition of movement. *Journal of Neurophysiology*, 114(2), 793–807. 25972588 10.1152/jn.00656.2014 PMC4533059

[R15] Korn C.W., Bach D.R. (2019). Minimizing threat via heuristic and optimal policies recruits hippocampus and medial prefrontal cortex. *Nature Human Behaviour*, 3(7), 733–745. 10.1038/s41562-019-0603-9 PMC6629544 31110338

[R16] Krawczyk D.C., D’esposito M. (2013). Modulation of working memory function by motivation through loss-aversion. *Human Brain Mapping*, 34(4), 762–74. 22113962 10.1002/hbm.21472 PMC3337893

[R17] Mobbs D., Petrovic P., Marchant J.L., et al. (2007). When fear is near: threat imminence elicits prefrontal-periaqueductal gray shifts in humans. *Science*, 317(5841), 1079–83. 17717184 10.1126/science.1144298 PMC2648508

[R18] Palminteri S., Khamassi M., Joffily M., Coricelli G. (2015). Contextual modulation of value signals in reward and punishment learning. *Nature Communications*, 6(1), 1–14. 10.1038/ncomms9096 PMC4560823 26302782

[R19] Payzan-LeNestour E., Bossaerts P., Behrens T. (2011). Risk, unexpected uncertainty, and estimation uncertainty: Bayesian learning in unstable settings. *PLoS Computational Biology*, 7(1), e1001048. 10.1371/journal.pcbi.1001048 PMC3024253 21283774

[R20] Penny W.D., Friston K.J., Ashburner J.T., Kiebel S.J., Nichols T.E. (Eds.) (2011). *Statistical Parametric Mapping: The Analysis of Functional Brain Images*. Elsevier.

[R21] Qi S., Hassabis D., Sun J., Guo F., Daw N., Mobbs D. (2018). How cognitive and reactive fear circuits optimize escape decisions in humans. Proceedings of the National Academy of Sciences, 115, 3186–91. 10.1073/pnas.1712314115 PMC5866541 29507207

[R22] Reddi B., Asrress K.N., Carpenter R.H. (2003). Accuracy, information, and response time in a saccadic decision task. *Journal of Neurophysiology*, 90(5), 3538–46. 12815017 10.1152/jn.00689.2002

[R23] Roy M., Shohamy D., Daw N., Jepma M., Wimmer G.E., Wager T.D. (2014). Representation of aversive prediction errors in the human periaqueductal gray. *Nature Neuroscience*, 17(11), 1607. 10.1038/nn.3832 PMC4213247 25282614

[R24] Schuck N.W., Gaschler R., Wenke D., et al. (2015). Medial prefrontal cortex predicts internally driven strategy shifts. *Neuron*, 86(1), 331–40. 25819613 10.1016/j.neuron.2015.03.015 PMC4425426

[R25] Sevgi M., Diaconescu A.O., Tittgemeyer M., Schilbach L. (2016). RETRACTED: social Bayes: using Bayesian modeling to study autistic trait–related differences in social cognition. *Biological Psychiatry*, 80(2), 112–19. 26831352 10.1016/j.biopsych.2015.11.025

[R26] Shenhav A., Botvinick M.M., Cohen J.D. (2013). The expected value of control: an integrative theory of anterior cingulate cortex function. *Neuron*, 79(2), 217–40. 23889930 10.1016/j.neuron.2013.07.007 PMC3767969

[R27] Shenhav A., Musslick S., Lieder F., et al. (2017). Toward a rational and mechanistic account of mental effort. *Annual Review of Neuroscience*, 40, 99–124. 10.1146/annurev-neuro-072116-031526 28375769

[R28] Simões‐Franklin C., Hester R., Shpaner M., Foxe J.J., Garavan H. (2010). Executive function and error detection: the effect of motivation on cingulate and ventral striatum activity. *Human Brain Mapping*, 31(3), 458–69. 19718655 10.1002/hbm.20879 PMC4485396

[R29] Stephan K.E., Penny W.D., Daunizeau J., Moran R.J., Friston K.J. (2009). Bayesian model selection for group studies. *Neuroimage*, 46(4), 1004–17. 19306932 10.1016/j.neuroimage.2009.03.025 PMC2703732

[R30] Tepper B.J., Moss S.E., Lockhart D.E., Carr J.C. (2007). Abusive supervision, upward maintenance communication, and subordinates’ psychological distress. *Academy of Management Journal*, 50(5), 1169–80.

